# Access and care issues in urban urgent care clinic patients

**DOI:** 10.1186/1472-6963-9-222

**Published:** 2009-12-04

**Authors:** David R Scott, Holly A Batal, Sharon Majeres, Jill C Adams, Rita Dale, Philip S Mehler

**Affiliations:** 1 Internal Medicine, Denver Health Medical Center, 777 Bannock Street, Denver, CO 80204, USA; 2 The Colorado Prevention Center, 789 Sherman Street, Suite 200, Denver, CO 80203, USA; 3 Oregon Health and Science University, 3181 SW Sam Jackson Park Road, Portland, OR 97239-3098 OP-30, USA

## Abstract

**Background:**

Although primary care should be the cornerstone of medical practice, inappropriate use of urgent care for non-urgent patients is a growing problem that has significant economic and healthcare consequences. The characteristics of patients who choose the urgent care setting, as well as the reasoning behind their decisions, is not well established. The purpose of this study was to determine the motivation behind, and characteristics of, adult patients who choose to access health care in our urgent care clinic. The relevance of understanding the motivation driving this patient population is especially pertinent given recent trends towards universal healthcare and the unclear impact it may have on the demands of urgent care.

**Methods:**

We conducted a cross-sectional survey of patients seeking care at an urgent care clinic (UCC) within a large acute care safety-net urban hospital over a six-week period. Survey data included demographics, social and economic information, reasons that patients chose a UCC, previous primary care exposure, reasons for delaying care, and preventive care needs.

**Results:**

A total of 1, 006 patients were randomly surveyed. Twenty-five percent of patients identified Spanish as their preferred language. Fifty-four percent of patients reported choosing the UCC due to not having to make an appointment, 51.2% because it was convenient, 43.9% because of same day test results, 42.7% because of ability to get same-day medications and 15.1% because co-payment was not mandatory. Lack of a regular physician was reported by 67.9% of patients and 57.2% lacked a regular source of care. Patients reported delaying access to care for a variety of reasons.

**Conclusion:**

Despite a common belief that patients seek care in the urgent care setting primarily for economic reasons, this study suggests that patients choose the urgent care setting based largely on convenience and more timely care. This information is especially applicable to the potential increase in urgent care volume in a universal healthcare system. Additionally, this study adds to the body of literature supporting the important role of timely primary care in healthcare maintenance.

## Background

Ideally, primary care should be the cornerstone of medical practice, but over the past decade there has been a trend towards increased utilization of urgent care clinics (UCCs) and emergency departments (EDs) for primary care needs [1,2]. Inappropriate use of urgent care infrastructure is recognized as a worldwide problem [3]. By virtue of its unique accessibility, the UCC in a public safety net hospital has developed an important and expanding role in the health care system. The UCC is considered a clinic independent of the primary care clinic setting and provides open access for patients seeking care for acute medical problems that are not severe enough to warrant Emergency Department evaluation. However, delivery of care via infrastructure geared towards acute, hospital-based services is problematic; patients who rely on these non-primary sources for health care may not receive preventive care services and fail to receive adequate care for their chronic medical conditions. Shea et al. demonstrated that patients who rely on an ED for blood pressure checks and medication refills are more likely to have severe uncontrolled hypertension [4]. The economic implications of seeking care in urgent care settings are also significant. A previously published National Medical Expenditure Survey estimated the annual cost difference between non-urgent ED visits compared to the cost of treatment in primary care facilities to be $1.3 billion dollars [5]. More worrisome is evidence that insurance coverage alone does not guarantee the use of timely and appropriate medical care [6,7].

Although the literature suggests many reasons why patients choose non-primary care clinics for their healthcare, there persists substantial uncertainty surrounding this patient population and the motivation driving their decision as to where to seek care [8]. Convenience has been cited to be a driving factor. Meditz et al. found that 37% of patients presenting for urgent care did so because they were off work and 20% because transportation was available at that time [9]. When asked to choose the most convenient time for a clinic appointment, these patients chose Saturday; the vast majority suggested that they would have chosen a primary care clinic over urgent care if it were open during the weekend or evening hours. Rizos et al. reported that the three most common reasons for attending walk-in clinics in Canada were convenient location, inability to see their regular physician soon enough, and that no appointment was needed in the urgent care setting [10]. Similarly, Shesser et al. found that patients tended to choose urban EDs because of convenience, the lack of a primary care provider, and the inability to make a prompt appointment with their regular provider [11]. In addition, patients seeking care in EDs equally cite lack of transportation and lack of insurance as the reason for not having a regular source of health care [12]. Interestingly, in one study, almost half the patients seeking care in an ED for a non-urgent complaint were referred there by a health care professional [13,14]. This may suggest that inadequate primary care infrastructure was playing as significant a role as inadequate patient insurance or education.

In the adult UCC at Denver Health Medical Center, the only public safety net hospital in Denver, the annual number of patient visits almost doubled between 1990 and 2000. The specific aim of this study was therefore to determine the motivation behind, and characteristics of, adult patients who choose to access health care in our UCC. Improving care to the primarily indigent patients Denver Health serves requires an understanding of why and how these patients access care. Simply providing increasing access to existing primary care facilities or providing universal health insurance will not rectify deficiencies in health care delivery unless the factors that compel patients to access urgent care are better understood.

## Methods

### Study Setting and Patients

This study was conducted at Denver Health Medical Center, an acute care safety-net urban hospital that is vertically integrated with a community health network of primary care clinics serving the residents of Denver, Colorado. The Walk-in Clinic is the hospital's adult UCC, which accepts patients from 7:30 AM until 10 PM seven days a week. Patients are seen in the UCC when they present directly for care, are referred from the ED, or referred from the hospital's network of community clinics. At the time of this study over 43, 000 patients were seen annually, the majority of whom were low-income, minority patients. Nearly half lacked medical insurance or obtained it through federally subsidized programs. Approximately 50% were Hispanic, 30% Caucasian, and 15% African-American. Over 20% of adults presenting for care spoke only Spanish.

### Study Protocol

All clinically stable patients presenting to the UCC between June 15 and August 11, 2000 who spoke either English or Spanish were eligible for study inclusion. Twenty percent of presenting patients were randomly selected for study participation through the use of a random number table, and selected patients were approached for study participation prior to their medical evaluation. The University of Colorado Institutional Review Board approved the study protocol and survey instrument, and written consent was obtained from all study subjects. The survey was administered to patients in either English or Spanish based on patient preference by research assistants fluent in both languages. Patients who presented to the clinic more than once during the study period were invited to participate in the survey one time only.

The questionnaire used in this study was developed specifically for this purpose but was based on prior questionnaires [12,15]. Piloting of the questionnaire was done prior to the study to determine its acceptability and to allow for an open-ended question format, in both English and Spanish, to ensure that the choice of reasons listed on the questionnaire for accessing care through the UCC was adequately inclusive. The questionnaire also included questions from the Medical Outcomes Study Short Form (SF-12) [16]. Items collected in the survey included level of education, primary language, insurance status, whether there was a phone in the home, availability of transportation, ability to read a newspaper, use of hospital facilities in the last year, reason for seeking care in the UCC, duration of the current health problem, whether the patient felt an immediate evaluation was necessary or could wait up to 24 hours for an appointment, whether the patient had attempted to get care for this problem elsewhere, whether the patient currently had a regular source of medical care and a primary care physician. Chart review was also performed by the research assistants to document insurance status, ethnicity, age, Denver residency, prior receipt of preventive care services within our health care system such as cholesterol testing, mammography, and Pap smear receipt, and the total number of clinic and hospital visits to the Denver Health system in the previous three years.

### Statistical Analysis

Population data were summarized using medians, ranges, frequencies and percentages. Multiple logistic regression models were used to identify factors associated with the following subpopulations: patients with no usual source of care, patients with no regular physician, and patients who delayed care for more than two days. Models were fit using backward selection and p < 0.1 as a cut off to keep independent variables in the models. Colinearity or correlation among independent variables was tested using Cronbach's Coefficient Alpha, and the Kappa Statistic. When independent variables showed colinearity the variable with the strongest association with the dependent variable on univariate analysis was used in the multiple logistic regression models [17]. Observations were excluded from the logistic regression analysis if either the dependent variable was missing or if any of the independent variables were missing. Interactions among the independent variables were not considered for the analysis. When a response was "don't know" or "decline to answer" or "N/A" it was considered missing. All analyses were done in SAS version 8.0 or higher.

## Results

During the time period of the study, there were 6, 564 patient visits made by 5, 497 patients. A total of 1, 262 patient visits were randomized to survey participation, representing 19.2% of all patient visits during the study period. Of those randomized, 256 patients (20.3%) refused participation, were ineligible, or had a previous clinic visit in which they were surveyed. The 1, 006 patients in the survey group represented 18.3% of all clinic patients seen during the study period. Although demographic information was not available for patients who refused to participate, surveyed patients were similar to the 5, 497 total clinic users during the study period in regards to age, ethnicity/race, gender, and insurance status (Table 1).

**Table 1:** Demographics of surveyed patients and all clinic patients during the study period

	Surveyed patients		All clinic patients	
	n = 1, 006	%	n = 5, 497	%
**Race/ethnicity**				
Hispanic	507	50.4%	2784	50.6%
White	301	29.9%	1633	29.7%
Black	162	16.1%	855	15.6%
Other	34	3.4%	225	4.1%
Missing	2	0.2%		
**Gender**				
Female	588	58.4%	3247	59.1%
Male	418	41.6%	2250	40.9%
**Insurance**				
No insurance*	723	71.9%	4090	74.4%
Medicaid	94	9.3%	652	11.9%
Medicare	44	4.4%	347	6.3%
Other	142	14.1%	408	7.4%
Missing	3	0.3%		
**Age in years**				
Median (range)	33 (18-90)		34 (18-97)	

*includes those on the state's indigent care program

Surveyed patients had many characteristics that reflected a lower socioeconomic status. 24.8% reported not having obtained a high school degree, 17.9% reported that they were unable to read a newspaper in English, and almost 12% were homeless. Twenty-five percent of respondents identified Spanish as their preferred language, and 23.6% of surveys were completed in Spanish.

**Figure 1:**
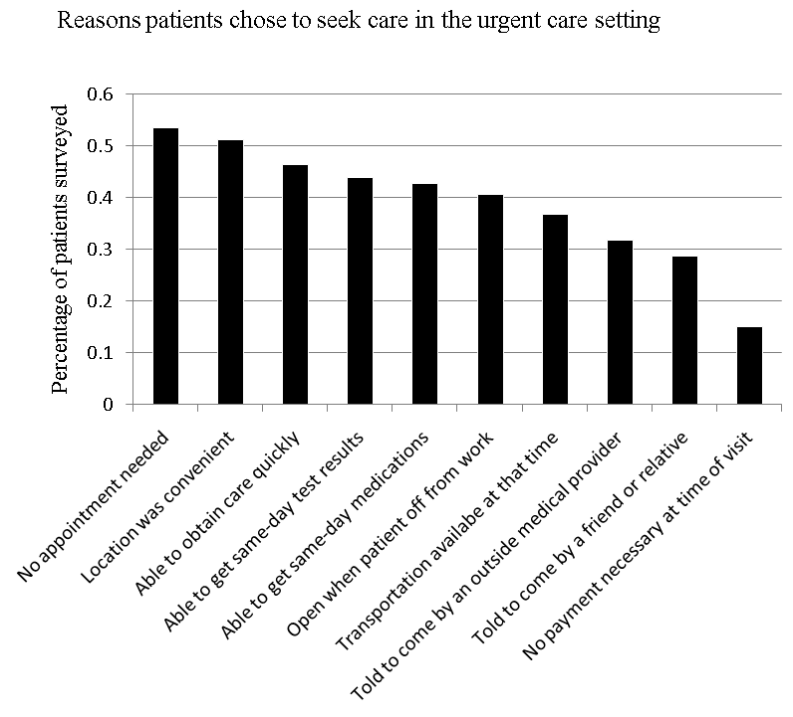
**Reasons patients chose to seek care in the urgent care setting**.

Patients were asked to note the reasons that they presented to the UCC; surveyed patients could respond positively to multiple reasons (Figure 1). 40.6% of patients reported they came to the UCC because it was open when they were off work, 36.9% because they had transportation available, 51.2% because the location was convenient, 53.5% because no appointment was necessary, 42.7% because they were able to get same day medications, and 43.9% because of same day test results. In addition, 46.4% noted that they came to the UCC because they could obtain care quickly. Notably, 31.9% of our surveyed patients reported that they had been told to come to the UCC by a physician or nurse, and 28.8% had been told to come to the UCC by a friend or relative. Only 15.1% of our patients reported that they chose to go to the UCC because co-payments were not mandatory.

Lack of a regular physician was reported by 67.9% of patients and 57.2% lacked a regular source of care. Results of multiple logistic regression analysis to determine predictors of the subpopulations are shown in Table 2. Those who were uninsured or participated in the state's indigent care program were more likely to lack a regular source of care (p < 0.01) and a regular physician (p < 0.01). Younger patients and male patients were also more likely to lack a regular source of care (p < 0.01) and a regular physician (p < 0.01). White patients were more likely than both black and Hispanic patients to lack a regular source of care (p = 0.04 and p = 0.008, respectively). Those who spoke Spanish as a primary language were more likely than primary English speakers to lack a regular physician (p < 0.0001).

**Table 2:** Factors associated with patients reporting no usual source of care and no regular physician

Predictor	Odds ratio	95% CI	p value
**No usual source of care (n = 575):**			
Male	2.3	1.68-3.08	< 0.001
Can not read English	1.9	1.22-3.03	0.005
White (vs Hispanic)	1.6	1.14-2.36	0.008
Not a resident of catchment area	1.6	1.04-2.54	0.032
Absence of a chronic medical condition	1.6	1.18-2.19	0.003
White (vs Black)	1.6	1.02-2.47	0.043
Not insured*	1.6	1.15-2.18	0.005
Transportation other than a car	1.5	1.06-2.09	0.021
Younger (decreasing by 10 years)	1.4	1.20-1.53	< 0.001

**No regular physician (n = 683):**			
Spanish as primary language	2.7	1.75-4.22	< 0.001
No phone available for use	2.3	1.02-5.32	0.046
Reports "can't afford MD appointment"	2.3	1.59-3.38	< 0.001
Not a resident of catchment area	2.2	1.36-3.73	0.002
Not insured*	2.2	1.59-3.08	< 0.001
Absence of a chronic medical condition	2.1	1.52-2.96	< 0.001
Male	2.0	1.44-2.75	< 0.001
Physical health or emotional problems don't interfere with social activities	1.7	1.19-2.46	0.004
White (vs Hispanic)	1.6	1.09-2.45	0.018
Younger (decreasing by 10 years)	1.3	1.18-1.51	< 0.001
White (vs Black)	1.1	0.66-1.70	0.811

*includes those on the state's indigent care program

There was a direct relationship in our patient population between receipt of preventive healthcare services and a regular source of health care other than an ED or UCC (data not shown). Patients who noted a regular source of care were significantly more likely to report having had a flu shot in the past twelve months (34.9% vs. 21.3%; p < 0.01) or having had a cholesterol test in the past five years (46.8% vs. 27.3%; p < 0.01). Chart review at our institution confirmed these self-reported results in regard to cholesterol testing in the past three years (24.9% vs. 7.6%; p < 0.01). Women who reported a regular source of care were significantly more likely to have had a documented Pap smear at our institution in the past three years (p < 0.01). However, even among those reporting a regular source of care, only 53.9% of women had a documented Pap smear in the past three years. Among women greater than age 50, 47.5% of those with a regular source of care had a documented mammogram in the past two years, compared to 36.4% of those without a regular source of care (p = 0.37).

Seventy-one percent of patients presenting for evaluation to our UCC had been sick for more than two days, with 49% noting that their current medical problem had been present for a week or longer. Factors that predicted a patient-reported delay of greater than two-days in seeking care are listed in Table 3. Those patients without insurance or on the state indigent care program were more likely to report a delay in seeking care. In addition, older patients, those who reported that their physical or emotional problems interfered with social activities, and those who were unable to afford physician appointments were more likely to delay seeking care. Among those who delayed care for more than two days, many reasons were reported: 26.4% identified lack of insurance, 50.1% were "not in enough pain until now", 45.9% were "not sick enough until now", 15.7% were unable to get off of work, 11.5% were unable to get transportation, and 9.8% were unable to contact their physician. Eighty-nine percent of patients felt that they needed to see a doctor immediately and 28.3% of patients had tried to see another doctor or been to another hospital previously with the same problem.

**Table 3:** Factors associated with patients reporting delaying care for two or more days (n = 713)

Predictor	Odds ratio	95% CI	p value
Saw a different physician first	2.0	1.40-2.83	< 0.001
Physical health or emotional problems interfered with social activities	1.5	1.02-2.20	0.040
Not insured*	1.4	1.04-2.01	0.028
Inability to afford physician appointments	1.4	0.98-1.97	0.064
Younger (decreasing by 10 years)	0.8	0.72-0.92	0.001
Not a resident of catchment area	0.7	0.46-1.02	0.061
No phone available for use	0.5	0.26-0.82	0.009

*includes those on the state's indigent care program

## Discussion

In this study we have documented many of the reasons that indigent patients rely on UCCs for their health care needs. The results confirm that patients tend to seek care in an urgent care setting due largely to convenience, affordability, and more timely care. Interestingly, the fact that there was no co-payment required at the time of the UCC visit was not reported as influential among a host of convenience factors. Our findings suggest that in order to shift a portion of the urgent care population to the primary care setting an attempt will not only need to be made to improve financial coverage for low income patients but also for the primary care setting to adopt many aspects of UCCs, which make them a preferred method to access care.

A perception of convenience was the most significant driving factor for patients in our study to seek care at the UCC. The lack of a need for an appointment was the most frequently cited reason (53.5%) for choosing urgent care. Since 40.6% of patients reported that they chose the UCC because it was open when they were off work we surmise that the long hours and 7 day schedule makes UCCs more accessible than primary care facilities which have more traditional hours. The degree to which the UCC's flexibility in scheduling attracted patients suggests that similar scheduling within conventional primary care systems may successfully attract a portion of these patients. The concept of "open access" appointment scheduling has been found to accommodate patients' urgent health care needs while providing continuous, routine care [18]. A study examining the implementation of an open access scheduling system within a large multispecialty medical group found patients with chronic diseases (depression, heart disease, or diabetes) decreased utilization of UCCs by roughly one-third when open access scheduling was available at the primary care clinic [19]. However, the study found no change in frequency of ED visits or hospitalizations.

In addition to suggesting convenience as a driving factor for patients choosing to obtain care at the UCC, our study also demonstrated that patients perceive the care in the urgent care setting to be timelier. Similar perceptions have previously been demonstrated as a driving factor for patients choosing urgent care [13]. Young et al. found that twenty-one percent of ambulatory patients seeking care in an ED did so because they felt that they would receive better care and/or more prompt diagnosis and treatment [13]. In our study, such perceptions were demonstrated by 43.9% of patients who recognized same-day test results as a primary reason for choosing our UCC and 46.4% who viewed the UCC as a means of obtaining care quickly. Notably, patient expectation of timely care has been shown to differ from that of physicians. In a recent study involving eleven written clinical scenarios, patients felt clinical evaluation should be performed significantly sooner than did physicians for eight of the eleven scenarios [20]. Acknowledging this discrepancy in perceptions and educating patients may help mitigate this rationale for choosing the urgent care setting.

The evidence from our study that patient decision making is largely driven by convenience and a desire for timely care is particularly applicable to the recent national debate surrounding universal health care and the restructuring of primary care. The need for convenient access and timely care delivery is one of the seven cornerstones of the new "patient-centered medical home [21]" as a model for primary care. Similarly, convenience and timely care have been emphasized in New England Journal of Medicine's recent perspective on "The Future of Primary Care" in which it is noted that "[p]atients needing urgent care must be able to get care on the day they request it [22]." The same article proposes that primary care should increasingly utilize "electronic or telephonic consultation" to serve as a convenient way of addressing patient issues while reducing demand for visits. Our data, which supports convenience as the largest driver in determining the means by which many patients seek care, strongly suggests that strategies such as these, which increase immediate healthcare accessibility, would be effective in attracting patients to the primary care setting.

The results of our study do raise some concern as to whether implementation of universal healthcare coverage without carefully addressing non-financial motivation for preferentially accessing urgent care may permit continued widespread miss-use of urgent care delivery systems. In particular, although lack of insurance was predictive of our patients delaying treatment and lacking primary care exposure, our data suggest non-economic causes to be equally strong predictors. Being male, white, or primarily Spanish speaking all had a higher odds ratio than did lack of insurance in predicting patient lack of a primary care source, and Spanish as a primary language had the highest odds ratio for predicting lack of a regular physician. Furthermore nearly three-quarters of our patients reported that not having to make a payment at the time of service was not perceived as an important reason for choosing the UCC. These findings suggest that increasing financial support, such as would be done through implementation of universal health coverage, may have only a limited effect on decreasing the number of non-urgent patients who present in the urgent care setting. This idea, in conjunction with a primary care shortage, can be seen in Massachusetts where the number of ED visits among low income newly insured patients remains 27% higher than the state average and concern exists as to whether overall urgent care visits may have actually increased since implementation of state-wide health insurance [23]. Similar trends in increased urgent care utilization have been predicted in models assessing the impact of health insurance on medical care utilization [24]. When viewed in the context of these examples our data strongly suggests a need for addressing the underlying social and cultural motivations highlighted in our study for choosing urgent care, in conjunction with offering wider financial support, to funnel non-urgent patients away from the urgent care setting.

Notably, our study also reinforced previous literature demonstrating that patients who preferentially seek care in the urgent care setting are at high risk of having many unmet preventive care needs. Our data demonstrated that patients with regular sources of primary care were significantly more likely to have had a flu shot, cholesterol test, or PAP smear within the generally accepted time frames. Access to primary care, even among those previously utilizing UCCs has been associated with decreased reliance on episodic care services and better quality of diabetes care [25].

An argument can be made that in light of the heavy reliance on UCCs by a large segment of the population that opportunities should be sought to provide preventive care interventions within the context of the UCC visit. Although follow-up remains problematic, we have previously shown it to be feasible with cervical cancer screening [26]. There are also similar data in providing other interventions, such as immunizations [27,28] without decreasing rates of subsequent use of primary care [29,30]. Our study's findings highlight the possibility of enhancing the receipt of these preventive services during UCC visits. Despite the benefit of offering preventive care services in an opportunistic manner via the urgent care setting, the literature has clearly demonstrated the importance of developing a stable patient-provider relationship, which is associated with better preventive services than is having a regular site of care [31].

There are several limitations to this study. Perhaps most importantly, patients surveyed were those seeking care in our UCC at least once during the study period, rather than a population-based study in which a random sample of community residents were interviewed about their urgent care needs. In addition, there was no demographic information available on survey nonrespondents, although comparison of the study patient population to all users of the clinic during the study period demonstrated similarity in age, race/ethnicity, gender, and insurance status. Because of the methodology used, we were only able to verify patients' self-reported prior health care use if it had occurred at our institution. We were also unable to objectively determine the urgency of a patient's presenting complaint and/or their need as perceived by health care professionals to access urgent care services. This study was conducted during the summer months, so there may be unique seasonal variations in care-seeking behaviors that cannot be analyzed here. We intentionally avoided conducting the study during the busy flu season because we knew it would be a confounder for accessing urgent care. In our health care system there is a standard method of triage between patients seeking care in the ED and UCC so that during the study period all urgent care appropriate patients were seen in the urgent care and eligible for study inclusion. However, we made no attempt to assess where the patient first attempted to seek care (presenting directly to the urgent care or triaged there from the ED); there may be substantial differences between these two patient populations that influenced our results. Lastly, our findings may not be generalizable to other populations with different patient and delivery system characteristics.

## Conclusion

The over utilization of urgent care has a negative impact on the economic sustainability of healthcare systems, as well as on the health of individual patients. The findings of this study offer an explanation as to why indigent patient populations are increasingly seeking care within an urgent care setting for non-urgent medical needs. Our results suggest that although this shift is partially in response to lack of insurance and financial limitations among patients, it is also driven by qualities attractive to patients, such as prompt diagnostic results and treatment, not needing an appointment, and flexible hours of operation. Understanding the motivation behind why patients seek care from UCCs is imperative in order to attempt to shift a portion of this population back to conventional primary care. A lack of proper health maintenance among this patient population is borne out in the literature and is supported by the results of this study. Possible strategies to address these concerns would be the adoption of attractive aspects of the urgent care setting within conventional primary care systems and the implementation of preventative care interventions within UCCs and EDs. Further research is recommended to determine patient response to such changes in the conventional primary care setting, as well as to further evaluate the efficacy of preventative care intervention within urgent care settings.

## Competing interests

The authors declare that they have no competing interests.

## Authors' contributions

HAB, SM, JCA, RD and PSM participated in the design and implementation of the study. RD performed the statistical analysis. DRS, HAB, RD, and PSM participated in interpretation of the study results helped to draft the manuscript. All authors read and approved the final manuscript

## Pre-publication history

The pre-publication history for this paper can be accessed here:

http://www.biomedcentral.com/1472-6963/9/222/prepub
